# Morphological features of larvae of *Drusus plicatus* Radovanović (Insecta, Trichoptera) from the Republic of Macedonia with molecular, ecological, ethological, and distributional notes

**DOI:** 10.3897/zookeys.598.7311

**Published:** 2016-06-14

**Authors:** Mladen Kučinić, Ana Previšić, Iva Mihoci, Vladimir Krpač, Ivana Živić, Katarina Stojanović, Ana Mrnjavčić Vojvoda, Luka Katušić

**Affiliations:** 1 Department of Biology (*Laboratory for Entomology), Faculty of Science, University of Zagreb, Rooseveltov trg 6, 10000 Zagreb, Republic of Croatia; 2 Croatian Natural History Museum, Demetrova 1, 10 000, Republic of Croatia; 3 Entomological Society for Investigation and Conservation of Biodiversity and sustainable Development of Natural Ecosystem, Vladimir Komarov st. 40b, 1000 Skopje, Republic of Macedonia; 4 University of Belgrade, Faculty of Biology, Belgrade, Republic of Serbia; 5 Croatian Centre for Agriculture, Food and Rural Affairs, Institute for Plant Protection, Rim 98, 10000 Zagreb, Republic of Croatia; 6 State Institute for Nature Protection, Radnička cesta 80, 10 000 Zagreb, Republic of Croatia

**Keywords:** Caddisfly, Drusinae, southeast Europe, larval description, fauna

## Abstract

A description of the larva of *Drusus plicatus* Radovanović is given for the first time. The most important diagnostic characters enabling separation from larvae of the other Drusinae from the southeast Europe are listed. Molecular, ecological, and ethological features and distribution patterns of the species are given. Additionally, information on the sympatric caddisfly species of the three springs where larvae and adults of *Drusus plicatus* were found and presented.

## Introduction

**Figure 1.**
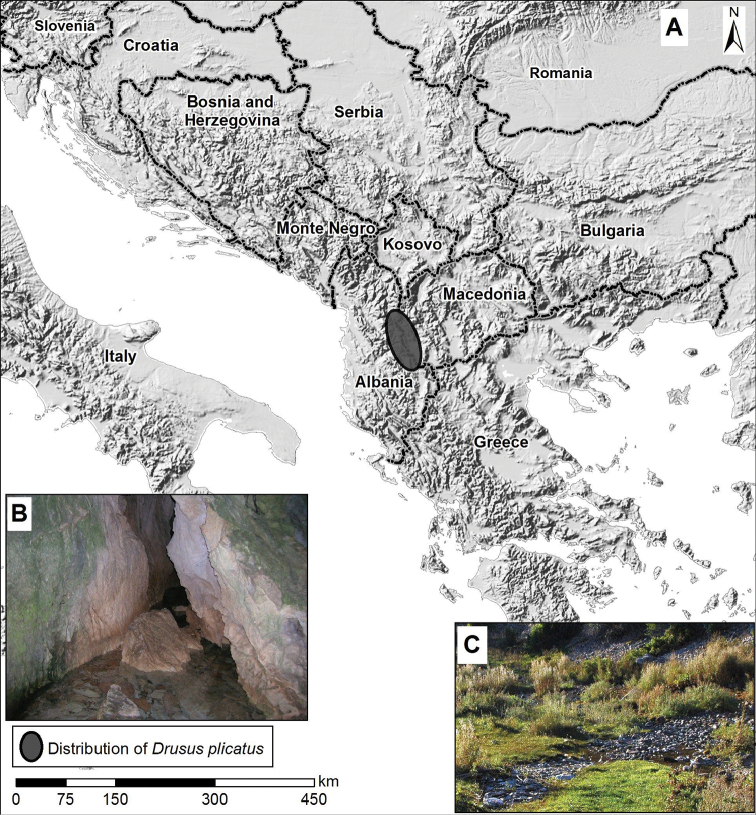
**A** Southeast Europe with distribution of *Drusus plicataus* (grey) **B** Spring Vevčani **C** Spring of the River Galička reka.

*Drusus plicatus* Radovanović (Limnephilidae, subfamiliy Drusinae), was described by Radovanović based on specimens collected in Labunište village situated in the southwest part of the Republic of Macedonia (Radovanović 1942) in southeast Europe (Fig. 1A). This region (southeast Europe) is delimited by the Croatia on the west and north, by the Serbia on the north, by the Bulgaria on the east and by the Greece on the south (Ecoregions: 5, 6, 7, 11, 12; Graf et al. 2008). In the area delimited in this way, 46 *Drusus* species have been recorded (e.g., Malicky 2004, 2005, Oláh 2010, 2011, Oláh and Kovács 2013, Kučinić et al. 2014, Ibrahimi et al. 2015, 2016, Vitecek et al. 2015a, 2015b, 2015c), from which six species are widely distributed (e.g., *Drusus biguttatus* Pictet, *Drusus chrysotus* Rambur, *Drusus croaticus* Marinković-Gospodnetić, *Drusus discolor* Rambur). The remaining 40 species are endemics of southeast Europe. Most species of *Drusus* from southeast Europe are reported from Bulgaria, Albania, Macedonia, and Bosnia and Herzegovina (e.g., Marinković-Gospodnetić 1979, Kumanski 1988, Malicky 2004, Oláh 2010, 2011, Oláh and Kovács 2013, Vitecek et al. 2015a, 2015b, 2015c), while the lowest number of species is recorded in Croatia (Kučinić et al. 2014). In recent years intensive research focussing on caddisfly diversity in southeast Europe has resulted in the description of 16 new species from the subfamily Drusinae (Oláh 2010, 2011, Oláh and Kovács 2013, Previšić et al. 2014a, Ibrahimi et al. 2015, 2016, Vitecek et al. 2015b, 2015c).

The subfamily Drusinae consists of eight genera with some 110 described species (Hickin 1967, Oláh 2010, 2011, Malicky 2004, Oláh and Kovács 2013, Oláh et al. 2015, Previšić et al. 2014a, Ibrahimi et al. 2015, 2016, Vitecek et al. 2015a, 2015b, 2015c), from which 95 belong to the genus *Drusus*, which is one of the largest genera in the European Trichoptera fauna (Malicky 2004).

Faunistic, phylogenetic, and phylogeographic characteristics of Drusinae have been studied extensively (e.g., Pauls et al. 2006, 2008, 2009; Previšić et al. 2009, 2012, 2014a, 2014b, Previšić and Popijač 2010, Ibrahimi et al. 2012, 2014, Stanić-Koštroman et al. 2012, 2015, Vitecek et al. 2015a). Additionally, taxonomic interest in the group was demonstrated by a number of studies focussing on the delineation of new species (Sipahiler 1992, Urbanič et al. 2002, Oláh 2010, 2011, Oláh and Kovács 2013, Previšić et al. 2014a, Ibrahimi et al. 2015, 2016, Vitecek et al. 2015b, 2015c) and larval taxonomy (e.g., Waringer et al. 2007, 2011, 2015, 2016, Kučinić et al. 2008, 2015, Vitecek et al. 2015a, 2015c). Larval morphology of all widely distributed species (e.g., *Drusus biguttatus*, *Drusus chrysotus*. *Drusus discolor*) of this genus recorded in southeast Europe is well known (Lepneva 1966, Waringer and Graf 1997, Previšić et al. 2012, Vitecek et al. 2015a); this is also valid for 16 of the southeast Europe endemic species (Kučinić et al. 2008, 2010, 2011a, 2011b, 2015, Vitecek et al. 2015a, 2015c, Waringer et al. 2015, 2016).

**Figure 2–4.**
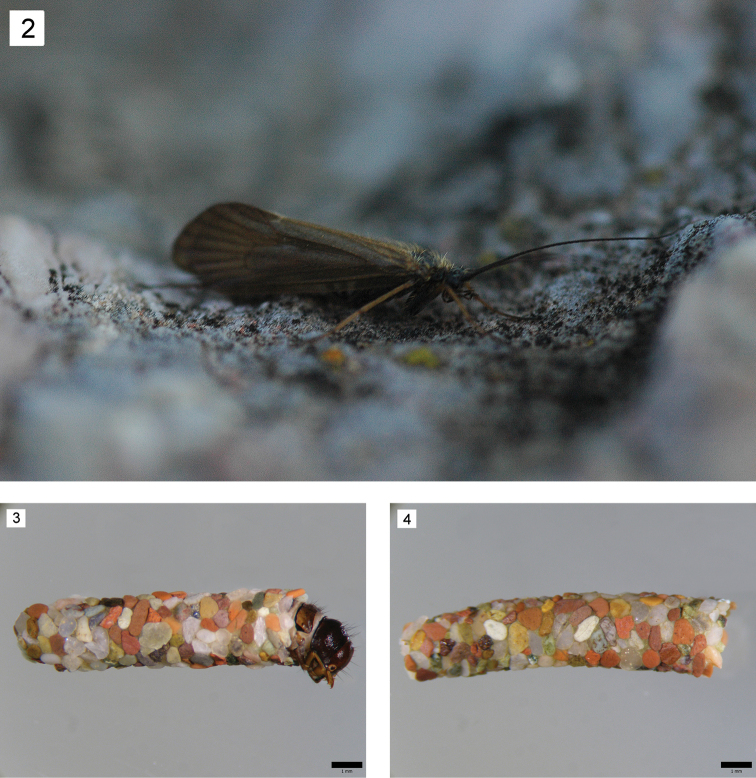
*Drusus plicatus* Radovanović. **2** Adult (spring of the River Galička reka) **3** Larva in case, 5th instar, right lateral view **4** Case, right lateral view.

The present study has three main objectives: 1. present the morphological features of the final larval instar of *Drusus plicatus*; 2. present molecular and ecological features and new data on the distribution of *Drusus plicatus*; 3. provide information on the caddisfly fauna in three springs in which larvae and adults of *Drusus plicatus* (Fig. 2) were found. Two of the springs are located in Mavrovo National Park, highlighting the importance of these data for the continued conservation of the protected areas of the Republic of Macedonia.

## Material and methods

### Fieldwork and sampling

The material studied comprises 7 larvae of *Drusus plicatus* collected on 23 August 2009 from the spring Vevčani (Fig. 1B), 12 larvae collected on 25 August 2009 (4th and 5th instar larvae), 24 larvae collected on 2 July 2010 (4th and 5th instar larvae), 4 larvae collected on 29 May 2013 from the spring of the River Galička reka (Fig. 1C), Mavrovo National Park, and 5 larvae of the same species collected on 2 July 2010 from the spring of the River Strežimirska reka, Mavrovo National Park (Table 1). Larvae were collected by handpicking and adults with an entomological net during the day. Collected specimens were stored in containers with 80% and 96% EtOH for morphological and molecular analysis, respectively.

**Table 1.** Sampling sites of *Drusus plicatus* in Republic of Macedonia and literature data of *Drusus plicatus* in Albania (Oláh and Kovács 2013).

Locality	Country	Altitude	Longitude (E)	Latitude (N)
Vevčani spring	Macedonia	ca 950 m	20.5844	41.2396
Modrič	Macedonia	ca 960 m	20.3425	41.2156
Tresonče	Macedonia	ca 1030 m	20.7223	41.5606
Mavrovska reka	Macedonia	ca 1290 m	20.4465	41.3843
spring of the river Galička reka	Macedonia	ca 1410 m	20.6646	41.5934
spring Sveta voda, Ničpur, river Radika	Macedonia	ca 980 m	20.4034	41.4435
spring - Rosočka Reka, Rosoki village	Macedonia	ca 1200 m	20.6933	41.5694
spring of River Reč	Macedonia	ca 1280 m	20.6348	41.7902
Mt Kaptinë, brooks	Albania	ca 1600 m	20.2889	41.3866
Cermenikë Mts, Zalli and Steblevës streams	Albania	ca 1270 m	20.4425	41.3083

Additionally, adult caddisfly communities in three springs in Macedonia (Vevčani spring, spring of the River Strežimirska reka, and the spring of the River Galička reka) were sampled using light traps. Identification of the adults was conducted using the works of Malicky (2004) and Kumanki (1988). The larval morphological terminology follows Wiggins (1996) and the systematics follow Morse (2015). Most of the collected specimens of larvae and adults are deposited in the collections of the first (Croatian Natural History Museum in Zagreb) and second authors (Faculty of Science, University of Zagreb). Some adults are deposited in the Macedonian Museum of Natural History in Skopje (collection Trichoptera Kučinić, Mihoci & Krpač).

We have included literature data for caddisfly species collected in Vevčani spring (*Rhyacophila trescavicensis* Botosaneanu, *Wormaldia occipitalis* Pictet, *Tinodes rostocki* McLachlan, *Ecclisopteryx keroveci* Previšić, Graf & Vitecek, *Potamophylax luctuosus* Piller & Mitterpacher) (Oláh and Kovács 2014) which were not found during our investigation of this spring.

### DNA extraction and PCR amplification

DNA was extracted from two adult males and two larvae of *Drusus plicatus* from the spring of the River Galička reka and one adult male and two larvae from Vevčani spring to confirm the association of the larvae with the adults. DNA extraction, amplification of the 541–bp–long fragment of the mitochondrial cytochrome oxidase I (mtCOI) using primers S20 and Jerry (Simon et al. 1994, Pauls 2004) were accomplished as outlined by Previšić et al. (2009). Sequences were edited manually using the program BioEdit v7.0.9 (Hall 1999) and aligned using ClustalX (Thompson et al. 1997). Sequences were deposited in GenBank under accession numbers listed in Table 2. Intraspecific *p*-distances were calculated using the software Mega 4.0.1 (Tamura et al. 2007).

**Table 2.** Intraspecific uncorrected pairwise distances (*p*) of partial mitochondrial cytochrome oxidase I (mtCOI) sequences observed in *Drusus plicatus* (shown as percent). Abbreviations are used to denote life stages; IM (M) = adult male, L = larva.

Locality	Specimen codes	Stage	DpMAIM1	DpMAIM2	DpMAL1	DpMAL2	DpVEIM1	DpVEL1	DpVEL2	GenBank accession nos
Spring of Galičnka reka, Mavrovo National Park	DpMAIM1	IM (M)								KT598014
	DpMAIM2	IM (M)	0.7							KT598015
	DpMAL1	L	0.7	0.0						KT598016
	DpMAL2	L	0.7	0.0	0.0					KT598017
Vevčani	DpVEIM1	IM (M)	1.1	1.1	1.1	1.1				KC881523
	DpVEL1	L	1.1	1.1	1.1	1.1	0.0			KT598018
	DpVEL2	L	0.6	0.9	0.9	0.9	0.9	0.9		KT598019

### Electron microscopy, macrophotography and biometry

Electron microscopy of larvae of *Drusus plicatus* (specimens from Vevčani spring) was carried out using a Tescan TS 5136 variable pressure scanning electron microscope (SEM). Samples were mounted with graphitic adhesive tape on the SEM stub and coated with carbon. The samples were examined by SEM operating in secondary electron (SE), or back-scattered (BSE) mode, at an accelerating voltage of 20 kV, running current of 110 pA, and variable pressure of 30 Pa to 5”10-1 Pa; sometimes the pressure was increased to 10 Pa to eliminate sample charging. Macrophotography and assessment of morphometric characteristics of pupae, larvae and larval cases were carried out using a Leica Wild MZ8 stereomicroscope and Olympus SP-500 UZ digital camera; photographs were processed with the software Olympus Quick Photo Camera 2.2. In the larvae of *Drusus plicatus* the following features were measured (in mm): head width, total body length, length of the anterior sclerites, their width at the widest median part and the distance between them, and also the length of the posterior sclerites. The following characters of cases were measured: total length, width of the anterior part, and width of the posterior part.

## Results

### Description of the fifth instar larva of *Drusus plicatus*

**Figure 5–7.**
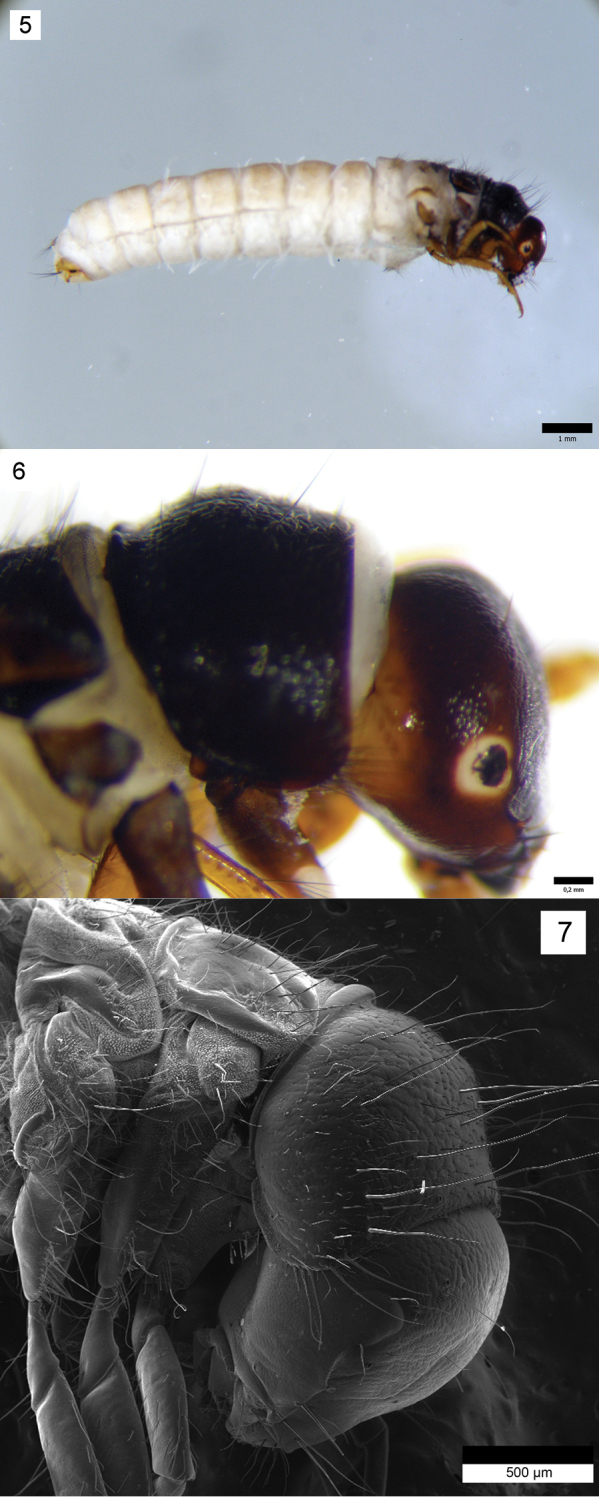
*Drusus plicatus* Radovanović, 5th instar larva. **5** Larva, right lateral view **6** Larva, head and pronotum, right lateral view **7** Head, pronotum and mesonotum, right lateral view.

Larval case constructed of mineral particles (Figs 3, 4), slightly curving, total length 9.97–19.19 mm, width of anterior part 2.30–2.70 mm, width of posterior part 1.64–2.01 mm. Overall body shape eruciform (Fig. 5).

**Figure 8–10.**
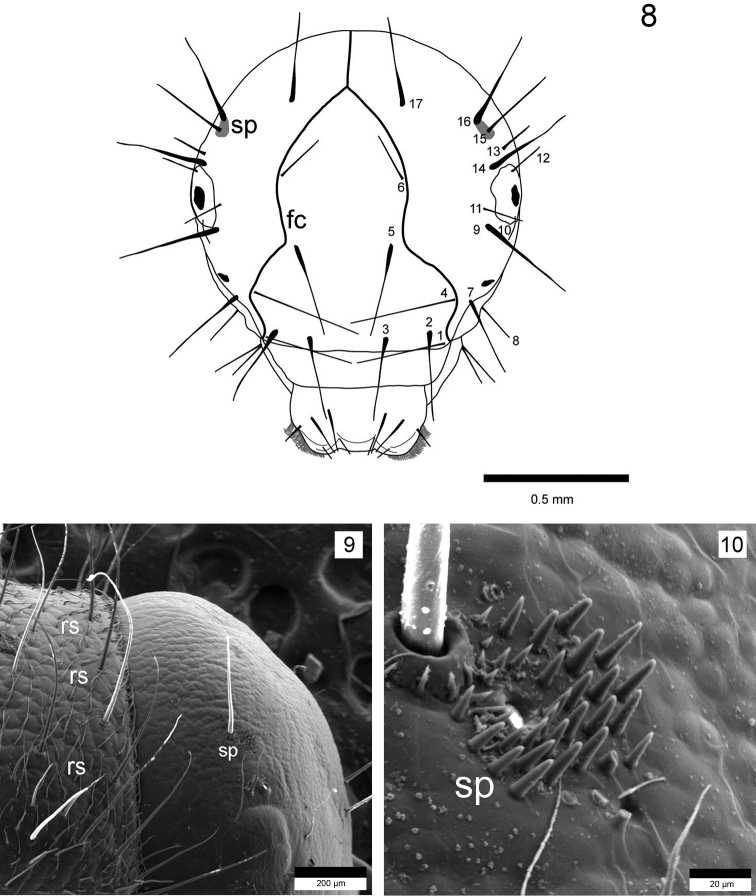
*Drusus plicatus* Radovanović, 5th instar larva. Head, fronotclypeus, and setae (with number), frontal view **9** Head, spinules (sp) and anterior part of pronotum with recumbent setae (rs), right lateral view **10** Spinules (sp) on the head, right lateral view.

**Figure 11–13.**
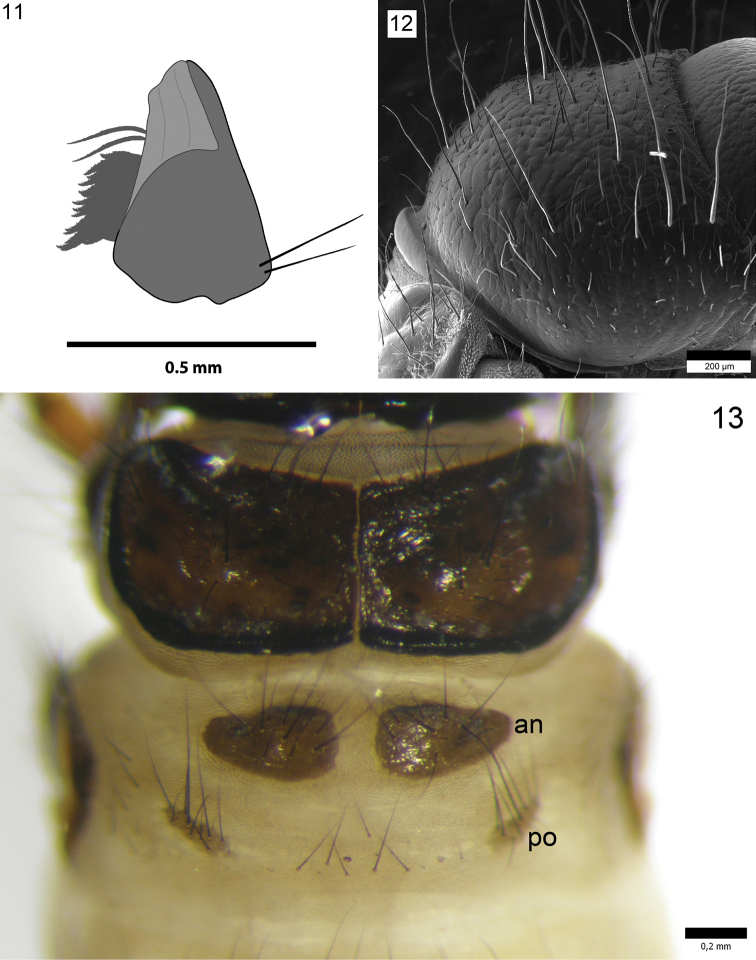
*Drusus plicatus* Radovanović, 5th instar larva. **11** Right mandible **12** Pronotum, right lateral view **13** Mesonotum, metanotum with anterior (an) and posterior sclerites (po), dorsal view.

Head capsule hypognathous (width 1.40–1.46 mm, n = 5) (Figs 5, 6, 7), in lateral view rounded in posterior dorsal part. Head (dark) brown to black, dorsally darker and laterally lighter (Fig. 6), with granular surface sculpturing and dark muscle attachment spots posteriorly. Genae reddish-brown to yellow with lighter (yellow) ring around each eye (Fig. 6). Frontoclypeal suture bell-shaped with narrow central region (Fig. 8). Antennae short, brown to dark brown (black), each positioned on small prominences (Fig. 6). Other primary setae positioned as shown in Fig. 8. Spinules (Figs 9, 10) present in small numbers, positioned around and between primary setae 15 and 16 (Fig. 8). Labrum symmetrical, brown to yellowish, with setal brush at anterolateral margins. Anterior part of labrum usually lighter. Mandibles black (Fig. 11), mesal part reddish. Typical for grazers, mesal margin with yellowish setal brush. Two setae present laterobasally on each mandible (Fig. 11). Labium and maxillae light-brown (yellowish). Each maxillary palp 5-segmented.

Thorax. Pronotum brown to black with granular surface sculpturing (Figs 6, 7, 12). Posterior margin rounded, both posterior and lateral margins thick and darkly sclerotized. In lateral view, anterior half of pronotum slightly concave, almost flat, posterior half slightly rounded (Figs 6, 7, 12). Pronotum bearing dark setae, especially laterally and on anterior margin, some of them long and conspicuous. Dorsal and lateral regions of pronotum bearing short, white, recumbent setae (Fig. 9).

Mesonotum sclerites brown, lighter than pronotum, with dark muscle attachment spots, dark setae and irregular surface (Fig. 13). Posterior and lateral margins thick and darkly sclerotized (Fig. 13).

Metanotum with 3 pairs of dorsal sclerites: anterior sclerites, posterior sclerites and lateral sclerites (Figs 5, 13). Anterior sclerites (*sa*1) elongated, triangular with rounded apices (Fig. 13), covered by setae, mainly in anterior part, color similar to mesonotum. Length of anterior sclerites 0.45–0.52 mm; width of anterior sclerites 0.25–0.31 mm; distance between anterior sclerites 0.07–0.11 mm. Posterior sclerites (*sa*2) smaller and lighter than *sa*1 sclerites (Fig. 13), triangularly or irregularly ellipsoid and with many setae. Length of posterior sclerites 0.26–0.31 mm. Lateral sclerites (*sa*3) (Fig. 5) longitudinally prolonged, sickle-shaped, lighter brown with dark median region, and group of setae anteriorly.

**Figure 14–18.**
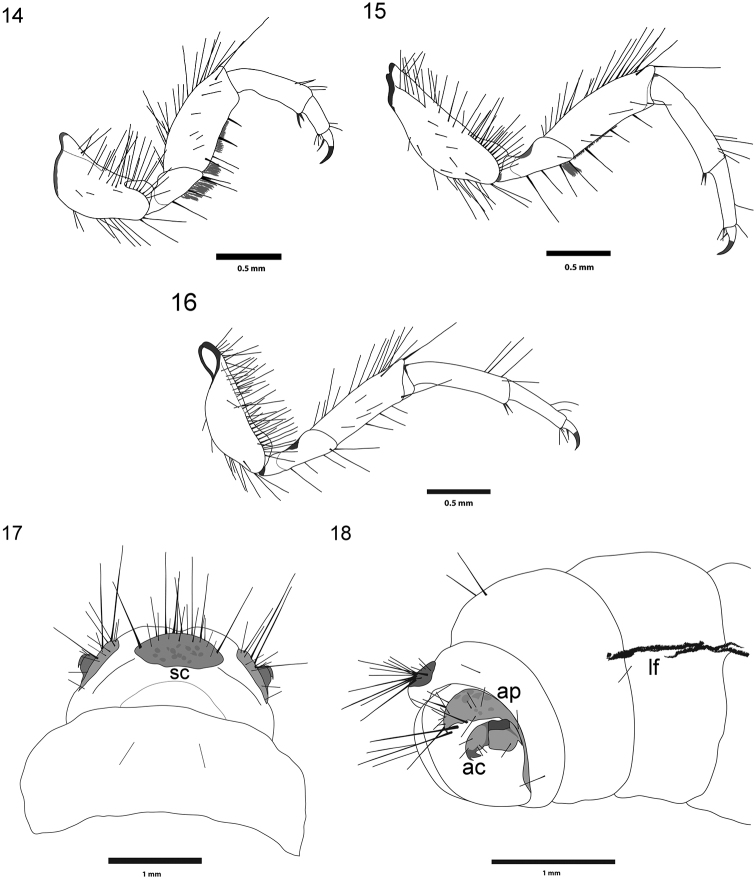
*Drusus plicatus* Radovanović, 5th instar larva. **14** Left foreleg **15** Left midleg **16** Left hindleg **17** Sclerites (sc) on segment IX, dorsal view **18** Anal proleg (ap), anal claws (ac), and lateral fringe (lf), right lateral view.

Legs (Figs 14, 15, 16) yellow-brown to brown or black, with dark ventral and dorsal margins. Foreleg coxae with dark setae on ventral and dorsal edges. Foreleg trochanters without dorsal setae, each with few light yellow setae on ventral margin, trochanteral brush present (Fig. 14). Mid- and hind leg coxae and femora (Figs 15, 16) with dark setae on both ventral and dorsal edges. Additional setae present on anterior and posterior faces of all femora. Setae on dorsal edges of tibiae present only distally on all legs. Foreleg coxae and femora wide compared to those of mid- and hind legs (Figs 14, 15, 16). Mid- and hind legs similar in shape and size (Figs 15, 16), with slender coxae, trochanters, femora and tibiae.

Abdomen. Abdominal segment I with well-developed dorsal and lateral humps (protuberances) with numerous ventral setae, some of them with small sclerites at bases. Lateral protuberances with few setae. Some of them (1-2) with small sclerites at bases. Single-filament gills (Fig. 5) present on segments II–VII. Lateral gills present on segments II-V (on segment V only pre-segmental gills are present). Lateral fringe extending from second half of segment III to first half of segment VIII (Fig. 18).

Segment IX bearing irregular, semicircular, light brown dorsal sclerite, with few long dark setae on posterior margin (Fig. 17). The anal prolegs typical of limnephilids (Fig. 18). Each with lateral sclerite longitudinally prolonged, sickle-shaped, yellowish, with small setae and 2 large, dark setae posteriorly (Fig. 18). Anal claws brown to dark brown.

### Ecology, ethology and distribution of *Drusus plicatus*

Mandible morphology of the larvae and observations during fieldwork suggest *Drusus plicatus* is a member of the Drusinae grazer clade (Previšić et al. 2014b). Species of this clade feed on epilithic algae and biofilms and can be found on stream bottoms, generally on cobbles, small pebbles and moss.

Based on the number of adults observed during the day, the most abundant population of *Drusus plicatus* was present in the spring of the River Galička reka (Fig. 1C). In this spring we observed two emergence peaks in spring and in late summer/autumn periods.

We collected *Drusus plicatus* larvae, adults or both in eight localities in the Republic of Macedonia (Table 1). Altitudes of locations where *Drusus plicatus* were collected range between approx. 950 m and 1410 m a.s.l. (Table 1).

### Sympatric caddisfly communities in three springs

We collected adult caddisflies at the three springs inhabited by *Drusus plicatus*. In the Vevčani spring the following species were recorded: *Rhyacophila balcanica* Radovanović, *Rhyacophila trescavicensis* (literature data), *Wormaldia occipitalis* (literature data), *Tinodes rostocki* (literature data), *Tinodes* sp. (female), *Ecclisopteryx keroveci* (literature data), *Drusus tenellus* Klapálek, *Drusus plicatus*, *Potamophylax latipennis* Curtis, *Potamophylax luctuosus* (literature data), in the spring of River Strežimirska reka: *Rhyacophila balcanica*, *Rhyacophila laevis* Pictet, *Synagapetus iridipennis* McLachlan, *Tinodes* sp. (female), *Hydropsyche* sp. (females), *Philopotamus montanus* Donovan, Annitella cf. triloba Marinković-Gospodnetić, *Drusus plicatus*, *Potamophylax pallidus* Klapálek, *Allogamus* sp. (male), *Thremma anomalum* Mclachlan and in the spring of the River Galička reka the following species: *Rhyacophila balcanica*, *Drusus plicatus*, *Philopotamus montanus*, *Thremma anomalum*, *Drusus plicatus* and *Potamophylax lemezes* Oláh & Graf.

## Discussion

### Association of larvae and adults of *Drusus plicatus*

Association of larvae and adults of *Drusus plicatus* is supported by the similarity of partial COI haplotypes. Since the association of larvae and adults is not completely reliable based solely on comparisons of sequences of a single gene from one specimen each (e.g., Zhou et al. 2007), we analysed specimens from two different populations. At each locality some adult males of *Drusus plicatus* and unassigned larvae shared identical COI haplotypes (Table 2). Observed variability in COI haplotypes within populations (Table 2) is in line with the variability of the same COI fragment in populations of some other *Drusus* species (e.g., Pauls et al. 2009, Previšić et al. 2009). Variability between populations in *Drusus plicatus* (Table 2), however, seems to be lower than observed in some other Dinaric *Drusus* endemics (e.g., *Drusus croaticus*, Previšić et al. 2009, *Drusus krusniki* Malicky, Previšić et al. 2014b).

Moreover, additional data, such as larvae and adults of *Drusus plicatus* recorded in 3 springs in Republic of Macedonia (Vevčani spring, spring of the River Galička reka, and the spring of the River Strežimirska reka), confirm our association of larvae and adults of *Drusus plicatus*. In these springs *Drusus plicatus* is sympatric with the following Drusinae species: *Drusus tenellus*, *Drusus botosaneanui* Kumanski and *Drusus biguttatus*, and larvae of these species exhibit different morphological characteristics from those observed in larvae of *Drusus plicatus* (Waringer and Graf 1997, Waringer et al. 2015).

### Separation of larvae of *Drusus plicatus* from other European Trichoptera larvae

Morphological features of the known larvae from the subfamily Drusinae are usually species specific and stable, enabling separation and identification of the species (e.g., Hickin 1967, Waringer and Graf 1997, Waringer et al. 2010, 2015). This is not the case for some other groups of Trichoptera in which larvae of many species are still not described or for which the separation of known larvae of some genera (e.g., *Hydroptila* Dalman, *Chaetopteryx* Stephens, *Rhyacophila* Pictet) is either very difficult or generally not possible (Waringer and Graf 1997).

Larvae from the subfamily Drusinae can be separated from other European Trichoptera larvae by the following morphological features (e.g., Waringer and Graf 1997, Graf et al. 2005, Kučinić et al. 2015): 1. A fully sclerotized pronotum and mesonotum; 2. Metanotum with six sclerites; 3. Gills with one filament; 4. Additional setae present on anterior and posterior faces of mid- and hind leg femora.

From the total of 49 Drusinae species recorded in southeast Europe, larval descriptions and taxonomic tools exist for the following 25 species: *Drusus balcanicus* Kumanski, *Drusus biguttatus*, *Drusus botosaneanui*, *Drusus bosnicus* Klapálek, *Drusus chrysotus* Rambur, *Drusus crenophylax* Graf & Vitecek, *Drusus croaticus*, *Drusus discolor*, *Drusus klapaleki* Marinković-Gospodnetić, *Drusus krpachi* Kučinić, Graf & Vitecek, *Drusus krusniki*, *Drusus macedonicus* Schmid, *Drusus medianus* Marinković-Gospodnetić, *Drusus meridionalis* Kumanski, *Drusus radovanovici* Marinković-Gospodnetić, *Drusus ramae* Marinković-Gospodnetić, *Drusus septentrionis* Marinković-Gospodnetić, *Drusus serbicus* Marinković-Gospodnetić, *Drusus siveci* Malicky, *Drusus tenellus*, *Drusus vernonensis* Malicky, *Drusus vespertinus* Marinković-Gospodnetić, *Ecclisopteryx dalecarlica* Kolenati, *Ecclisopteryx ivkae* Previšić, Graf & Vitecek and *Ecclisopteryx keroveci* (Kučinić et al. 2008, 2010, 2011a, 2011b, 2015, Previšić et al. 2014a, Vitecek et al. 2015a, 2015c, Waringer et al. 2010, 2015, 2016).

*Drusus plicatus* larvae can be easily distinguished from larvae of these species by the following morphological features:

– *Drusus chrysotus*, *Drusus discolor*, *Drusus krpachiDrusus meridionalis* and *Drusus siveci* have mandibles with terminal teeth and filtering bristles on legs and the first abdominal sternite, *Drusus plicatus* does not have any of the listed morphological features;

– *Drusus chrysotus*, *Drusus discolor*, *Drusus krpachi*, *Drusus meridionalis* and *Drusus siveci* have a head capsule concavity, a typical characteristic for larvae of these species, which is absent in *Drusus plicatus* larvae;

– Larvae of *Drusus plicatus*, *Drusus bosnicus* and *Drusus ramae* differ in head capsule shapes in lateral view. In *Drusus bosnicus* and *Drusus ramae* the head vertex is flat, while in *Drusus plicatus* the vertex is slightly rounded;

– *Drusus ramae* has a specific shape of the pronotum with two prominent acute humps on the posterior part, while the posterior part of the pronotum in *Drusus plicatus* is rounded; *Drusus plicatus* has areas of spinules on the head capsule that are absent in *Drusus ramae*;

– Larvae of *Drusus bosnicus*, *Drusus klapaleki*, *Drusus krusniki*, *Drusus medianus*, *Drusus septentrionis* and *Drusus vespertinus* have a pronounced hump in the central part of the pronotum in lateral view which is absent in *Drusus plicatus*, in which the pronotum is flat in the anterior part and slightly rounded in the posterior part;

– Larvae of *Drusus serbicus* have a recognizable shape of the pronotum in lateral view with an annular crest highest at dorsal center and gradually declining laterally, while the pronotum of *Drusus plicatus* larvae has a different shape (flat in the anterior part and slightly rounded in the posterior part);

– Larvae of *Drusus serbicus* lack lateral gills on the abdomen, *Drusus plicatus* has lateral gills on abdominal segments II throughout V;

– Larvae of *Drusus croaticus* lack prominent, long median setae dorsally on the anterior border of the pronotum and spinule areas on the head, which can be found in *Drusus plicatus*;

– Larvae of *Drusus radovanovici* and *Drusus vernonensis* have the dorsal part of the pronotum covered with numerous thin long, yellow (yellowish) setae, which are lacking in *Drusus plicatus*;

– Larvae of *Drusus botosaneanui*, *Drusus tenellus*, *Ecclisopteryx dalecarlica*, *Ecclisopteryx ivkae* and *Ecclisopteryx keroveci* have distinct parietal spines on the head, which are absent in *Drusus plicatus*;

– The whole pronotum of *Drusus plicatus* larvae is covered in white recumbent setae, *Drusus crenophylax* lacks these setae in a semicircular area anterior to the pronotal ridge, *Drusus biguttatus* generally lacks these recumbent setae on the whole pronotum;

– Larvae of *Drusus balcanicus* and *Drusus biguttatus* lack spinule areas on the head, which can be found in *Drusus plicatus*.

**Figure 19.**
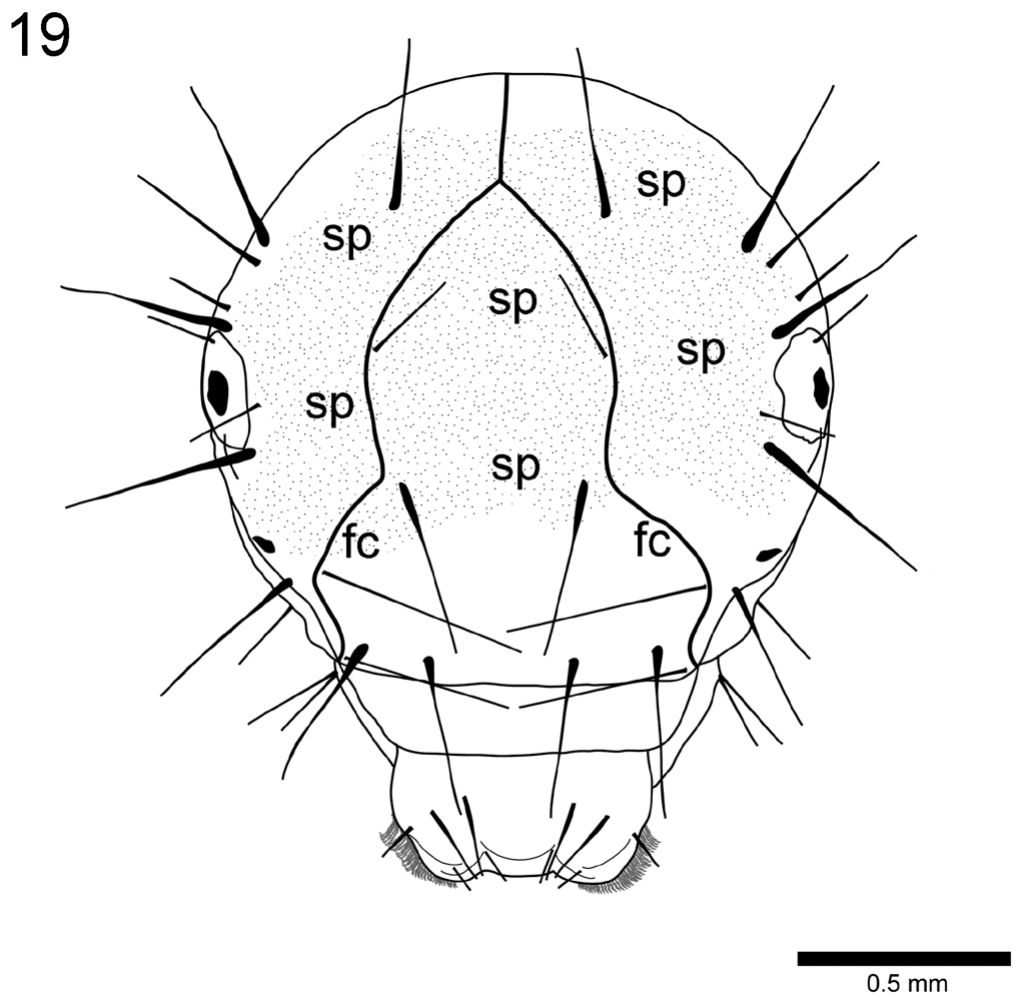
*Drusus plicatus* Radovanović, 4th instar larva. Head, frontoclypeus (fc) and area with spinules (sp), frontal view.

Interestingly, the last larval instar of *Drusus plicatus* differs from the earlier larval stages not only in head capsule width, but also in the larger extent of spinule fields (Fig. 19, fourth instar larva). So far, this feature was noticed only for the earlier larval stages of *Drusus bosnicus* (M. Kučinić, unpublished data) and for last instars of *Drusus vernonensis* (Waringer et al. 2016).

**Figures 20–21.**
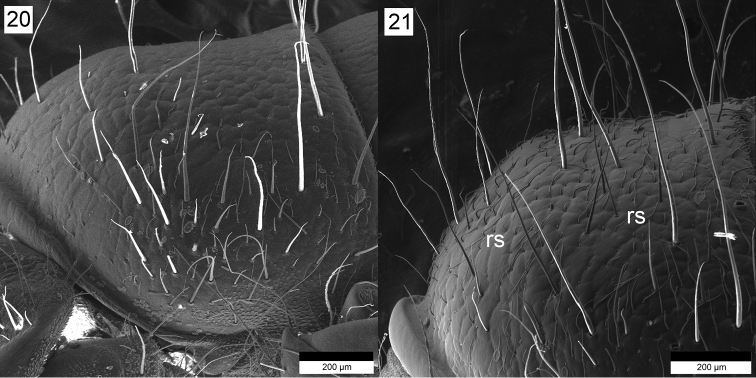
**20** *Drusus biguttatus* Pictet. Pronotum, right lateral view **21** *Drusus plicatus* Radovanović. Pronotum, showing recumbent setae (rs), right lateral view.

Faunistic research conducted in western Macedonia, for the last eight years recoverd besides *Drusus plicatus*, eight more species from the genus *Drusus*: *Drusus biguttatus*, *Drusus vernonensis*, *Drusus botosaneanui*, *Drusus discolor*, *Drusus discophorus* Radovanovic, *Drusus macedonicus*, *Drusus krpachi* and *Drusus tenellus* (Radovanović 1942, Botosaneanu 1960, Vitecek et al. 2015a, 2015b, Waringer et al. 2015, 2016). From all the above listed species only larva of *Drusus discophorus* was not described yet. Of these species only *Drusus biguttatus* and *Drusus plicatus* larvae cannot be easily distinguished (Figs 20, 21). Differentiation of *Drusus biguttatus* larvae from *Drusus plicatus* larvae can be done by careful examination of morphological features on the pronotum (Figs 20, 21) and on the head.

*Drusus discophorus* larvae have not been described yet, but this species seem to be limited to the type locality consists of a spring and little mountain stream at Labuniško Lake (Jablanica Mt.). In this locality we never found larvae or adults of *Drusus plicatus* during several years of repeated collections. Radovanović described both species from the Jablanica Mt. and stated that *Drusus discophorus* inhabits higher elevations (1900 m a.s.l.), while *Drusus plicatus* inhabits lower altitudes (approx. up to 900 m a.s.l., Labunište village) (Radovanović 1942). In this investigation we recorded *Drusus plicatus* in localities at higher elevation (approx. 1410 m a.s.l., spring of the River Galička reka), and Oláh and Kovács (2013) found this species in one location in Albania at an elevation of approx. 1600 m a.s.l. (Table 1). However, the morphology of male genitalia of *Drusus plicatus* and *Drusus discophorus* is very similar (Radovanović 1942, Malicky 2004), and a comprehensive study using morphology and molecular genetic data is necessary to enable clear separation of all stages of these two species.

### Ecological and ethological aspects and distribution of *Drusus plicatus*

Based on shared morphological (dark coloring of the imago, morphology of genitalia), and behavioral features (diurnal activity), *Drusus plicatus* could be closely related to the *Drusus bosnicus* group that is represented by a great number of species in southeast Europe (Marinković-Gospodnetić 1976, 1978, Kučinić et al. 2014, Vitecek et al. 2015c). Most *Drusus bosnicus* group species exhibit highly similar male genital morphology (Mariković-Gospodnetić 1978, Malicky 2004, Kučinić et al. 2011a, 2011b, Vitecek et al. 2015c). Analysis of the molecular data of *Drusus plicatus*, as well as of the other *Drusus* species (Malicky 2004, 2005, Oláh 2010, 2011, Oláh and Kovács 2013, Kučinić et al. 2014, Vitecek et al. 2015c), could show which species belong to the *Drusus bosnicus* group and clarify their phylogenetic and evolutionary relationships.

The subfamily Drusinae has been shown to comprise 3 groups differing in larval feeding ecology and morphology (Pauls et al. 2008). Also, these groups represent distinct evolutionary lineages (Pauls et al. 2008; Vitecek et al. 2015a). Based on the morphology of the larvae mandibles of *Drusus plicatus* are grazers. In addition to species with grazing larvae (e.g., species from *Drusus bosnicus* group, *Drusus plicatus*) (Kučinić et al. 2014, Viteck et al. 2015c), southeast Europe, along with western Alps, is a center of diversity for species with different larval feeding behaviors, for example, carnivorous filters (*Drusus meridonalis*, *Drusus macedonicus*, *Drusus krpachi*, *Drusus siveci*) (Vitecek et al. 2015a, 2015b). The mandibles of grazers are morphologically different from larvae that have carnivorous filtering feeding behavior (Pauls et al. 2008, Kučinić et al. 2011a, 2011b, 2015, Vitecek et al. 2015a). Molecular data from grazers and carnivorous filterers indicate a closer phylogenetic relationship among species in each group and also suggest certain evolutionary processes of speciation that probably happened in the ancestors of each feeding group (Marinković-Gospodnetić 1978, Kučinić et al. 2011a, Pauls et al. 2008, Vitecek et al. 2015a). Data suggest greater similarity for species that are geographically closer and have a similar feeding behaviour (Previšić et al. 2014b, Vitecek et al. 2015a) with *Drusus plicatus* grouping with grazers from Albania, for example *Drusus arbanios* Oláh, *Drusus dacothracus* Oláh, *Drusus illyricus* Oláh and *Drusus pelasgus* Oláh (Previšić et al. 2014b). Speciation of these and other *Drusus* is driven not only by the allopatric distribution caused by distinct geological and hydrological processes (e.g., karstification) in the past (Previšić et al. 2014b), but also by specific biologies that also condition this type of distribution, such as limited dispersal ability of adults (Kučinić et al. 2014, Geismar et al. 2015).

According to Schmid (1956), species of the *Drusus bosnicus* group are distributed in southeast Europe and the Alps. Generally, all are endemics or micro-endemics with small distribution areas and known only one or a few populations per species (Marinković-Gospodnetić 1979, Kučinić et al. 2008, Oláh 2010, 2011, Oláh and Kovács 2013, Vitecek et al. 2015c). *Drusus krusniki* is an exception, as more populations of this species are known (Previšić et al. 2014b). We collected *Drusus plicatus* at 8 localities in the Republic of Macedonia and the species is further reported from two localities in Albania (Oláh and Kovács 2013) (Table 1), rendering this also one of the more widely distributed endemic *Drusus bosnicus* group species in the southeast of Europe. We did not find *Drusus plicatus* at the type locality in Labunište village (Radovanović 1942), but we collected larvae and adults of this species in Vevčani spring (Table 1, Fig. 1B), several kilometres from Labunište village. Type locality in Labunište village was destroyed by anthropogenic influence: high level of urbanisation, pollution, stream canalisation.

The distance between the southern-most (Vevčani spring) (Fig. 1B) and the northern-most sampling location (spring of the River Strežimirska reka) of *Drusus plicatus* is about 100 km (Fig. 1A). Compared to the other species of the *Drusus bosnicus* group in the southeast Europe, this is a relatively large distance (Marinković-Gospodnetić 1978, 1979, Kučinić et al. 2014).

*Drusus plicatus* inhabits the creanal zone of streams and rivers with adults day-active at or near the spring. Diurnal activity is reported for several *Drusus* species in southeast Europe, e.g., *Drusus krusniki*, *Drusus vespertinus*, *Drusus medianus*, *Drusus klapaleki*, *Drusus radovanovici* (Kučinić et al. 2014, M. Kučinić, A. Previšić, unpublished data). However, a small number of *Drusus plicatus* specimens were collected also during the night using UV light traps at the spring of the River Galička reka, which is an exception for dark colored species of caddisflies that generally are active during day (Kučinić et al. 2014). At this locality, the highest abundance of *Drusus plicatus* has been recorded, with several hundreds of adults, during the day.

A similar mass emergence of adults has been previously recorded in *Drusus septentrionis* at two localities in Bosnia and Herzegovina (springs of the rivers Bistrica and Sturba, Kučinić et al. 2008, M. Kučinić, unpublished data) and in *Drusus krusniki* at Alipaša’s springs in Montenegro (A. Previšić unpublished data). We observed two peaks in the emergence of *Drusus plicatus* at the spring of the River Galička reka, the first one in spring (May - June) and the second one in autumn (September). The same emergence pattern was recorded for some other *Drusus* species in the Balkan Peninsula, e.g. *Drusus croaticus* and *Drusus septentrionis* (Kučinić 2002, Kučinić et al. 2008).

### Caddisfly species richness

Among the three springs encompassed in this study the highest biodiversity (species richness) of caddisflies was recorded in the spring of the River Strežimirska reka, and the lowest in the spring of the River Galička reka. Only two species, *Rhyacophila balcanica* and *Drusus plicatus*, were recorded in all three springs. Also during this study, *Synagapetus iridipennis* was recorded for the first time for the Trichoptera fauna of the Republic of Macedonia.

*Potamophylax lemezes* was described based on specimens collected in the spring of the River Galička reka (Oláh et al. 2013). The exact taxonomic status of this population would ideally be assessed using molecular methods for a comparison of this population with some other populations of *Potamophylax nigricornis* Pictet, from which *Potamophylax lemezes* was delineated (Oláh et al. 2013).

According to the literature *Wormaldia occipitalis* was recorded from Vevčani spring (Oláh and Kovács 2014). During our investigation we did not collect specimens of any *Wormaldia* from this locality. The taxonomic status of this species will be evaluated in future studies following Neu (2015), because this species is not present in the Republic of Macedonia.
